# Supplementary material for: A Likelihood Approach for Real-Time Calibration of Stochastic Compartmental Epidemic Models
Source: PLoS Comput Biol. 2017 Jan 17;13(1):e1005257. doi: 10.1371/journal.pcbi.1005257 (PMC5240920; doi:10.1371/journal.pcbi.1005257)
Supplement: S1 File — (TAR.GZ) [file pcbi.1005257.s014.tar.gz › HSPH_Online-SI-Revision/Additions/MSS11/n100-extreme-SEITR_noisy/n100-extreme-SEITR_noisy_NEW_table0.pdf]

1	R0	{1.38654, 9.85565, 0.1}	{2.93978, 19.9817, 0.}	{{2.93978, 19.9817, 0.}, {2.93978, 19.9817, 0.}}	{112.022, 102.744, 100.}	inf	18653.	27970.6	{27815.4, 28123.7}	49.9521	0.175948	newly	12047.4	9262.79	{9152., 9372.}	23.1135	0.114168	Reff	0.312221	0.747941	{0.744306, 0.751064}	139.555	ar	92379.	93600.9	{93467., 93723.}	1.32266
2	R0	{2.36629, 12.2029, 0.1}	{2.80387, 10.6536, 0.}	{{2.80387, 10.6536, 0.}, {2.80387, 10.6536, 0.}}	{18.4921, 12.6961, 100.}	inf	35008.	25448.	{25257.9, 25652.3}	27.308	0.13852	newly	17453.	14702.5	{14542., 14842.}	15.7593	0.0744927	Reff	0.202507	0.692356	{0.688108, 0.696843}	241.892	ar	99022.	92535.9	{92389., 92709.}	6.55021
3	R0	{2.32253, 12.836, 1.}	{1.96378, 4.99867, 0.}	{{1.88643, 4.56746, 0.}, {2.17292, 6.16453, 0.}}	{15.4466, 61.0574, 100.}	inf	36298.	13201.7	{11302.1, 17883.}	63.6297	0.448216	newly	18266.	15037.	{13840., 17814.}	17.6778	0.087109	Reff	0.29092	0.791277	{0.755747, 0.879568}	171.991	ar	98997.	78302.9	{75969., 83957.}	20.9038
4	R0	{1.79075, 11.2058, 1.}	{2.04387, 6.609, 0.}	{{1.9862, 6.28417, 0.}, {1.9862, 6.28417, 0.}}	{14.1349, 41.0216, 100.}	inf	27988.	14887.7	{13810.6, 14280.7}	46.807	0.280572	newly	16022.	13641.9	{13277., 13801.}	14.8553	0.0701103	Reff	0.341174	0.804376	{0.797252, 0.810636}	135.767	ar	96920.	80260.1	{78973., 79755.}	17.1894
5	R0	{2.26952, 9.62371, 0.1}	{2.77394, 8.24535, 0.}	{{2.77394, 8.24535, 0.}, {2.77394, 8.24535, 0.}}	{22.2258, 14.3225, 100.}	inf	29454.	22376.5	{22184.2, 22537.6}	24.0291	0.119361	newly	16920.	15408.	{15270., 15586.}	8.93629	0.0406676	Reff	0.133902	0.569346	{0.565685, 0.572892}	325.197	ar	98881.	92258.4	{92107., 92393.}	6.69756
6	R0	{1.57191, 6.89004, 0.1}	{2.99092, 10.3743, 0.}	{{2.99092, 10.3743, 0.}, {2.99092, 10.3743, 0.}}	{90.2727, 50.5697, 100.}	inf	21085.	26395.	{26194., 26617.8}	25.1837	0.0975394	newly	17405.	15212.8	{15061., 15377.}	12.5951	0.0584796	Reff	0.240125	0.608398	{0.602831, 0.612495}	153.367	ar	95046.	93976.4	{93845., 94107.}	1.12536
7	R0	{1.99226, 17.2339, 1.}	{2.88523, 18.2127, 0.}	{{2.88523, 18.2127, 0.}, {2.88523, 18.2127, 0.}}	{44.8219, 5.67928, 100.}	inf	30023.	26666.3	{26500.2, 26856.4}	11.1804	0.0514966	newly	11332.	9566.68	{9442., 9684.}	15.5782	0.073566	Reff	0.270071	0.70053	{0.696971, 0.703497}	159.387	ar	97992.	93227.9	{93089., 93365.}	4.86173
8	R0	{2.63369, 15.1879, 0.1}	{2.59708, 9.39342, 0.}	{{2.59708, 9.39342, 0.}, {2.59708, 9.39342, 0.}}	{1.39001, 38.1519, 100.}	inf	36804.	21798.1	{21545.3, 22003.8}	40.7724	0.227488	newly	14766.	13945.9	{13796., 14107.}	5.55411	0.0248333	Reff	0.159786	0.657373	{0.65306, 0.661754}	311.409	ar	99451.	90452.6	{90283., 90639.}	9.04804
9	R0	{2.53271, 9.90552, 0.1}	{2.77394, 8.24535, 0.}	{{2.77394, 8.24535, 0.}, {2.77394, 8.24535, 0.}}	{9.52454, 16.76, 100.}	inf	31361.	22358.4	{22202.2, 22527.4}	28.7064	0.146956	newly	17411.	15404.1	{15278., 15535.}	11.5267	0.0531992	Reff	0.11382	0.569714	{0.565277, 0.574694}	400.539	ar	99355.	92253.8	{92113., 92386.}	7.14725
10	R0	{2.48791, 18.9003, 2.}	{2.39277, 11.1504, 0.98}	{{2.38758, 11.1331, 1.}, {2.38758, 11.1331, 1.}}	{4.0803, 41.0042, 51.}	inf	38471.	21344.1	{21071.5, 21493.6}	44.519	0.25597	newly	13614.	12506.	{12300., 12650.}	8.13853	0.0369075	Reff	0.296509	0.863902	{0.85846, 0.868887}	191.357	ar	99291.	87722.9	{87391., 87911.}	11.6507
11	R0	{2.84414, 18.4765, 1.}	{2.52588, 12.2207, 0.}	{{2.52588, 12.2207, 0.}, {2.52588, 12.2207, 0.}}	{11.1899, 33.858, 100.}	inf	41946.	23494.5	{23295.1, 23692.8}	43.9887	0.251734	newly	14675.	12709.7	{12579., 12851.}	13.3923	0.0624621	Reff	0.221814	0.892308	{0.888102, 0.896664}	302.277	ar	99654.	89594.3	{89410., 89801.}	10.0947
12	R0	{2.29369, 14.622, 0.1}	{2.13505, 8.31782, 0.}	{{2.13505, 8.31782, 0.}, {2.13505, 8.31782, 0.}}	{6.91644, 43.1144, 100.}	inf	33245.	15995.3	{15861.5, 16136.7}	51.8866	0.317746	newly	14159.	11877.7	{11728., 12033.}	16.1121	0.0763204	Reff	0.188656	0.757602	{0.754245, 0.760619}	301.579	ar	98982.	83004.3	{82716., 83259.}	16.142
13	R0	{2.73413, 16.7529, 1.}	{2.9884, 12.8326, 0.}	{{2.9884, 12.8326, 0.}, {2.9884, 12.8326, 0.}}	{9.29971, 23.4007, 100.}	inf	39057.	28290.5	{28136.9, 28457.6}	27.5662	0.140064	newly	14704.	13960.5	{13827., 14093.}	5.05679	0.0225505	Reff	0.173699	0.722082	{0.717203, 0.727054}	315.708	ar	99543.	93948.6	{93838., 94066.}	5.62012
14	R0	{1.79554, 16.5943, 2.}	{2.56757, 17.005, 0.}	{{2.56757, 17.005, 0.}, {2.56757, 17.005, 0.}}	{42.9973, 2.47477, 100.}	inf	28239.	24097.2	{23917.8, 24251.6}	14.667	0.0688892	newly	11642.	9558.06	{9435., 9675.}	17.9002	0.0856792	Reff	0.390135	0.894274	{0.890511, 0.897936}	129.222	ar	96821.	90109.5	{89931., 90308.}	6.93184
15	R0	{1.91429, 13.7771, 1.}	{2.88523, 18.2127, 0.}	{{2.88523, 18.2127, 0.}, {2.88523, 18.2127, 0.}}	{50.7205, 32.1952, 100.}	inf	28640.	26662.4	{26481.6, 26818.9}	6.90508	0.0310789	newly	13230.	9554.69	{9412., 9659.}	27.7801	0.141363	Reff	0.264038	0.700691	{0.69704, 0.704374}	165.375	ar	97541.	93208.5	{93086., 93349.}	4.44172
16	R0	{1.42094, 10.5871, 2.}	{2.15677, 10.1363, 0.}	{{2.15677, 10.1363, 0.}, {2.15677, 10.1363, 0.}}	{51.7849, 4.25818, 100.}	inf	20384.	17583.	{17412.8, 17780.7}	13.7413	0.0642105	newly	12953.	11359.	{11210., 11509.}	12.3063	0.0570542	Reff	0.417131	0.873889	{0.870452, 0.877505}	109.5	ar	93044.	83440.9	{83168., 83696.}	10.321
17	R0	{1.45515, 6.16466, 0.1}	{1.9208, 4.75911, 0.}	{{1.88643, 4.56746, 0.}, {2.17292, 6.16453, 0.}}	{32.0004, 22.8001, 100.}	inf	20742.	12260.	{11286.7, 17629.5}	40.8931	0.233484	newly	20574.	14477.6	{13784., 17737.}	29.6315	0.154124	Reff	0.356002	0.773419	{0.754187, 0.869127}	117.251	ar	93254.	77156.6	{75981., 83396.}	17.2618
18	R0	{2.85669, 13.2416, 0.1}	{2.9194, 10.1535, 0.}	{{2.9194, 10.1535, 0.}, {2.9194, 10.1535, 0.}}	{2.19533, 23.3211, 100.}	inf	38339.	26344.3	{26161.9, 26510.7}	31.286	0.16296	newly	17189.	15645.6	{15497., 15785.}	8.97911	0.0408691	Reff	0.132179	0.652565	{0.649002, 0.655893}	393.698	ar	99644.	93473.4	{93345., 93600.}	6.19269
19	R0	{2.56417, 17.9558, 0.1}	{2.15677, 10.1363, 0.}	{{2.15677, 10.1363, 0.}, {2.15677, 10.1363, 0.}}	{15.8881, 43.5487, 100.}	inf	39573.	17582.4	{17410.7, 17765.4}	55.5698	0.352334	newly	14542.	11355.1	{11211., 11534.}	21.9149	0.107456	Reff	0.282084	0.874119	{0.870671, 0.877588}	209.879	ar	99389.	83429.1	{83156., 83665.}	16.058
20	R0	{2.27517, 11.1864, 0.1}	{2.99092, 10.3743, 0.}	{{2.99092, 10.3743, 0.}, {2.99092, 10.3743, 0.}}	{31.459, 7.25957, 100.}	inf	31962.	26414.6	{26228., 26589.4}	17.3561	0.082796	newly	16864.	15217.4	{15065., 15372.}	9.76417	0.0446354	Reff	0.172117	0.607468	{0.602966, 0.612223}	252.94	ar	98894.	93993.2	{93845., 94126.}	4.95562
21	R0	{2.35735, 10.4735, 0.1}	{2.77394, 8.24535, 0.}	{{2.77394, 8.24535, 0.}, {2.77394, 8.24535, 0.}}	{17.6719, 21.2741, 100.}	inf	33381.	22392.9	{22200.4, 22582.3}	32.9172	0.173398	newly	18543.	15426.5	{15266., 15577.}	16.8069	0.0799248	Reff	0.172039	0.569066	{0.564813, 0.572667}	230.776	ar	99036.	92262.9	{92112., 92392.}	6.83902
22	R0	{1.79551, 11.9179, 2.}	{2.13505, 8.31782, 0.}	{{2.13505, 8.31782, 0.}, {2.13505, 8.31782, 0.}}	{18.9104, 30.2074, 100.}	inf	26731.	15992.1	{15835.7, 16157.}	40.174	0.223125	newly	14003.	11858.8	{11722., 12004.}	15.3123	0.0722001	Reff	0.270045	0.757433	{0.753156, 0.761149}	180.484	ar	97033.	82986.5	{82741., 83251.}	14.476
23	R0	{1.23545, 6.31687, 0.1}	{2.26582, 8.12562, 0.}	{{2.26582, 8.12562, 0.}, {2.26582, 8.12562, 0.}}	{83.4007, 28.6336, 100.}	inf	13943.	15831.8	{15681.1, 15991.1}	13.5465	0.0551593	newly	13260.	11439.4	{11314., 11566.}	13.7299	0.0641585	Reff	0.306775	0.63482	{0.631343, 0.638557}	106.934	ar	88739.	85638.	{85436., 85875.}	3.49455
24	R0	{2.24089, 10.1789, 0.1}	{2.93906, 10.3908, 0.}	{{2.93906, 10.3908, 0.}, {2.93906, 10.3908, 0.}}	{31.1558, 2.08158, 100.}	inf	33782.	27390.8	{27141.7, 27592.8}	18.9188	0.0910887	newly	20012.	16147.6	{15997., 16301.}	19.3103	0.0931949	Reff	0.242442	0.701501	{0.696113, 0.706625}	189.348	ar	98868.	93609.3	{93481., 93760.}	5.31896
25	R0	{1.57298, 10.2607, 0.1}	{2.52588, 12.2207, 0.}	{{2.52588, 12.2207, 0.}, {2.52588, 12.2207, 0.}}	{60.5796, 19.1023, 100.}	inf	23805.	23474.1	{23262.6, 23672.5}	1.3925	0.00609925	newly	15457.	12691.8	{12531., 12832.}	17.8896	0.08562	Reff	0.403627	0.892199	{0.88772, 0.896842}	121.046	ar	94957.	89600.9	{89415., 89786.}	5.64056
26	R0	{1.2154, 8.2576, 0.1}	{2.7499, 15.3124, 0.}	{{2.7499, 15.3124, 0.}, {2.7499, 15.3124, 0.}}	{126.255, 85.4342, 100.}	inf	15080.	25518.7	{25354.4, 25673.5}	69.2219	0.228452	newly	11730.	10844.7	{10713., 10973.}	7.54697	0.034098	Reff	0.35796	0.753769	{0.750421, 0.756771}	110.574	ar	88421.	92036.3	{91891., 92176.}	4.08873
27	R0	{2.22841, 18.7983, 2.}	{2.7499, 15.3124, 0.}	{{2.7499, 15.3124, 0.}, {2.7499, 15.3124, 0.}}	{23.4018, 18.5436, 100.}	inf	34057.	25533.9	{25372.6, 25684.1}	25.0261	0.125095	newly	11908.	10855.9	{10743., 10955.}	8.83515	0.0401866	Reff	0.242317	0.753844	{0.750377, 0.757409}	211.098	ar	98756.	92052.2	{91878., 92205.}	6.78822
28	R0	{1.27778, 5.36767, 1.}	{1.88643, 4.56746, 0.}	{{1.88643, 4.56746, 0.}, {1.88643, 4.56746, 0.}}	{47.633, 14.9079, 100.}	inf	16522.	11471.4	{11265.7, 11695.8}	30.5687	0.15849	newly	18063.	14006.	{13791., 14205.}	22.4602	0.110511	Reff	0.340107	0.759771	{0.755103, 0.764722}	123.392	ar	89843.	76241.6	{75864., 76601.}	15.1391
29	R0	{2.38042, 15.8901, 2.}	{2.2983, 8.81055, 0.}	{{2.2983, 8.81055, 0.}, {2.2983, 8.81055, 0.}}	{3.45001, 44.5532, 100.}	inf	37211.	19264.5	{19062.6, 19427.5}	48.229	0.285926	newly	15388.	13690.4	{13535., 13845.}	11.0319	0.0507821	Reff	0.307407	0.796065	{0.791649, 0.800452}	158.961	ar	99112.	86238.5	{85968., 86463.}	12.9888
30	R0	{2.53188, 12.7607, 0.1}	{2.17292, 6.16453, 0.}	{{2.17292, 6.16453, 0.}, {2.17292, 6.16453, 0.}}	{14.1778, 51.6913, 100.}	inf	39222.	17883.9	{17662.9, 18043.1}	54.4035	0.341087	newly	19690.	17855.2	{17608., 18066.}	9.31823	0.0425014	Reff	0.319143	0.877576	{0.87176, 0.883544}	174.979	ar	99317.	83791.9	{83529., 84035.}	15.6319
31	R0	{1.24482, 10.5471, 1.}	{2.39327, 14.7451, 0.}	{{2.29055, 13.7413, 0.}, {2.93978, 19.9817, 0.}}	{92.2584, 39.8027, 100.}	inf	15788.	20274.8	{18627.5, 27852.1}	28.4187	0.104442	newly	9931.	8979.65	{8806., 9248.}	9.5796	0.0438191	Reff	0.364384	0.76535	{0.750683, 0.781035}	110.039	ar	89097.	87376.9	{85866., 93492.}	3.1547
32	R0	{2.30446, 17.9607, 1.}	{2.73634, 13.3345, 1.}	{{2.73634, 13.3345, 1.}, {2.73634, 13.3345, 1.}}	{18.7411, 25.7575, 0.}	inf	35756.	25436.2	{25251.4, 25597.2}	28.8618	0.147903	newly	13001.	12287.8	{12161., 12397.}	5.48542	0.024516	Reff	0.270705	0.76337	{0.759241, 0.767325}	181.994	ar	99028.	91898.7	{91750., 92026.}	7.19924
33	R0	{2.31579, 11.8236, 2.}	{1.94705, 4.66877, 0.}	{{1.94705, 4.66877, 0.}, {1.94705, 4.66877, 0.}}	{15.9228, 60.5131, 100.}	inf	30363.	10849.9	{10668.3, 11006.1}	64.2661	0.446947	newly	15166.	12350.4	{12140., 12498.}	18.5651	0.089215	Reff	0.14124	0.67115	{0.666936, 0.674993}	375.184	ar	99012.	78186.4	{77847., 78475.}	21.0334
34	R0	{1.6678, 10.6744, 1.}	{2.71225, 12.7047, 1.}	{{2.71225, 12.7047, 1.}, {2.71225, 12.7047, 1.}}	{62.6243, 19.0204, 0.}	inf	23352.	23182.2	{23009.7, 23349.1}	0.773766	0.00337842	newly	13645.	11381.9	{11250., 11492.}	16.5853	0.0787734	Reff	0.259643	0.640178	{0.636146, 0.644121}	146.561	ar	95809.	91696.2	{91552., 91858.}	4.29272
35	R0	{1.65476, 7.07559, 0.1}	{2.77394, 8.24535, 0.}	{{2.77394, 8.24535, 0.}, {2.77394, 8.24535, 0.}}	{67.6339, 16.5324, 100.}	inf	23549.	22367.	{22155.8, 22547.7}	5.01935	0.0223736	newly	19217.	15409.5	{15248., 15553.}	19.8132	0.0959091	Reff	0.270404	0.568759	{0.564572, 0.572856}	110.337	ar	95746.	92262.4	{92093., 92420.}	3.63842
36	R0	{2.71807, 18.5959, 0.1}	{2.22376, 9.35432, 0.}	{{2.22376, 9.35432, 0.}, {2.22376, 9.35432, 0.}}	{18.1862, 49.6969, 100.}	inf	41864.	18939.	{18787.6, 19122.5}	54.7608	0.344495	newly	15024.	13295.9	{13167., 13448.}	11.5021	0.0530811	Reff	0.322961	0.91648	{0.912631, 0.920821}	183.774	ar	99540.	84837.6	{84585., 85091.}	14.7704
37	R0	{2.6202, 12.7552, 0.1}	{2.96079, 12.6717, 0.}	{{2.9884, 12.8326, 0.}, {2.9884, 12.8326, 0.}}	{13.9816, 1.81955, 100.}	inf	39131.	27925.6	{28028., 28468.3}	28.6357	0.147631	newly	18951.	13943.5	{13765., 14081.}	26.4233	0.133277	Reff	0.212734	0.725243	{0.717851, 0.728609}	240.915	ar	99426.	93649.5	{93804., 94099.}	5.80986
38	R0	{1.47672, 9.81651, 1.}	{2.52368, 12.5037, 0.}	{{2.52368, 12.5037, 0.}, {2.52368, 12.5037, 0.}}	{70.8975, 27.3743, 100.}	inf	20846.	21821.	{21641.1, 22004.1}	4.67728	0.0198441	newly	13535.	11104.3	{10991., 11248.}	17.9587	0.085984	Reff	0.299449	0.722259	{0.718655, 0.725557}	141.196	ar	93815.	89538.3	{89351., 89700.}	4.55865
39	R0	{1.90004, 12.3313, 1.}	{2.13505, 8.31782, 0.}	{{2.13505, 8.31782, 0.}, {2.13505, 8.31782, 0.}}	{12.3686, 32.5471, 100.}	inf	28756.	15979.3	{15800.6, 16160.7}	44.4313	0.255184	newly	15042.	11853.3	{11702., 11981.}	21.199	0.103489	Reff	0.303056	0.757215	{0.752961, 0.761075}	149.86	ar	97413.	82982.3	{82693., 83223.}	14.8139
40	R0	{2.64218, 18.5237, 2.}	{2.99939, 13.7076, 1.}	{{2.99939, 13.7076, 1.}, {2.99939, 13.7076, 1.}}	{13.5196, 25.9995, 50.}	inf	37865.	27339.6	{27111.7, 27517.9}	27.7972	0.141452	newly	13194.	12529.2	{12381., 12677.}	5.03903	0.0224745	Reff	0.17626	0.648804	{0.64503, 0.652532}	268.095	ar	99474.	94040.1	{93928., 94160.}	5.46266
41	R0	{1.6945, 7.17996, 0.1}	{2.17292, 6.16453, 0.}	{{2.17292, 6.16453, 0.}, {2.17292, 6.16453, 0.}}	{28.2334, 14.1425, 100.}	inf	26451.	17888.8	{17672.1, 18101.1}	32.37	0.16988	newly	23349.	17857.4	{17601., 18068.}	23.5197	0.11647	Reff	0.379415	0.87711	{0.87105, 0.882466}	131.174	ar	96332.	83813.	{83470., 84089.}	12.9956
42	R0	{1.84559, 18.0074, 1.}	{2.03032, 10.7564, 0.}	{{2.02235, 10.6804, 0.}, {2.02235, 10.6804, 0.}}	{10.0093, 40.2971, 100.}	inf	28741.	14922.9	{14630.1, 14954.}	48.0779	0.285679	newly	10541.	9015.62	{8893., 9156.}	14.4709	0.0679412	Reff	0.338389	0.81956	{0.816053, 0.821813}	142.195	ar	97358.	80367.4	{79892., 80578.}	17.4517
43	R0	{2.24965, 17.0361, 2.}	{2.85364, 13.5122, 0.}	{{2.86001, 13.5542, 0.}, {2.86001, 13.5542, 0.}}	{26.8714, 20.6847, 100.}	inf	35312.	27924.6	{27803.8, 28163.7}	20.9205	0.102227	newly	14237.	13652.4	{13500., 13794.}	4.10599	0.0182236	Reff	0.339337	0.880814	{0.875511, 0.885804}	159.569	ar	98770.	92899.9	{92798., 93153.}	5.9432
44	R0	{2.54324, 15.7967, 0.1}	{2.13505, 8.31782, 0.}	{{2.13505, 8.31782, 0.}, {2.13505, 8.31782, 0.}}	{16.0501, 47.3446, 100.}	inf	36249.	15987.7	{15797.4, 16166.9}	55.8947	0.355525	newly	14170.	11858.2	{11688., 12010.}	16.3151	0.0773791	Reff	0.167193	0.757466	{0.753844, 0.76133}	353.05	ar	99354.	83022.8	{82737., 83245.}	16.4374
45	R0	{2.54943, 14.8032, 0.1}	{2.59708, 9.39342, 0.}	{{2.59708, 9.39342, 0.}, {2.59708, 9.39342, 0.}}	{1.8691, 36.5447, 100.}	inf	36278.	21783.3	{21600.3, 22009.6}	39.9546	0.221531	newly	14996.	13933.	{13756., 14112.}	7.08829	0.0319474	Reff	0.179352	0.657394	{0.652536, 0.661745}	266.538	ar	99380.	90449.4	{90283., 90619.}	8.98631
46	R0	{2.37755, 12.6461, 2.}	{1.88643, 4.56746, 0.}	{{1.88643, 4.56746, 0.}, {1.88643, 4.56746, 0.}}	{20.6568, 63.8824, 100.}	inf	36257.	11487.4	{11297.9, 11699.5}	68.3168	0.499214	newly	17991.	14011.	{13792., 14207.}	22.1222	0.108623	Reff	0.261673	0.759322	{0.75349, 0.764269}	190.18	ar	99096.	76266.	{75925., 76586.}	23.0383
47	R0	{1.69279, 11.6636, 1.}	{2.52588, 12.2207, 0.}	{{2.52588, 12.2207, 0.}, {2.52588, 12.2207, 0.}}	{49.2143, 4.77662, 100.}	inf	25788.	23488.3	{23303.2, 23680.2}	8.91774	0.0405755	newly	14547.	12693.5	{12558., 12840.}	12.7415	0.059209	Reff	0.3499	0.891649	{0.887163, 0.895803}	154.83	ar	96214.	89614.1	{89422., 89757.}	6.85963
48	R0	{1.82402, 10.0038, 1.}	{2.70861, 10.3614, 0.}	{{2.70795, 10.355, 0.}, {2.70795, 10.355, 0.}}	{48.4966, 3.57459, 100.}	inf	27920.	25207.1	{24990.1, 25378.8}	9.71653	0.0444058	newly	17877.	15294.6	{15125., 15487.}	14.4456	0.0677739	Reff	0.343281	0.779004	{0.773147, 0.78305}	126.929	ar	97076.	91633.1	{91432., 91806.}	5.60685
49	R0	{1.6424, 11.1667, 2.}	{2.71225, 12.7047, 1.}	{{2.71225, 12.7047, 1.}, {2.71225, 12.7047, 1.}}	{65.1393, 13.7732, 50.}	inf	22603.	23160.7	{22992.7, 23324.}	2.46727	0.0105777	newly	12499.	11375.1	{11230., 11511.}	8.99216	0.0409405	Reff	0.249793	0.640329	{0.635976, 0.643573}	156.344	ar	95546.	91689.8	{91529., 91860.}	4.03595
50	R0	{2.05638, 14.3048, 1.}	{2.75598, 12.4651, 1.}	{{2.75598, 12.4651, 1.}, {2.75598, 12.4651, 1.}}	{34.0209, 12.8608, 0.}	inf	31592.	25547.3	{25356.7, 25709.8}	19.1337	0.0922385	newly	14245.	13053.1	{12906., 13177.}	8.36687	0.0379594	Reff	0.290299	0.74839	{0.744203, 0.75308}	157.8	ar	98194.	92090.7	{91927., 92245.}	6.21552
